# Automatic segmentation of brain MRI using a novel patch-wise U-net deep architecture

**DOI:** 10.1371/journal.pone.0236493

**Published:** 2020-08-03

**Authors:** Bumshik Lee, Nagaraj Yamanakkanavar, Jae Young Choi

**Affiliations:** 1 Department of Information and Communications Engineering, Chosun University, Gwangju, Republic of Korea; 2 Division of Computer & Electronic Systems Engineering, Hankuk University of Foreign Studies, Yongin-si, Republic of Korea; University of Alberta, CANADA

## Abstract

Accurate segmentation of brain magnetic resonance imaging (MRI) is an essential step in quantifying the changes in brain structure. Deep learning in recent years has been extensively used for brain image segmentation with highly promising performance. In particular, the U-net architecture has been widely used for segmentation in various biomedical related fields. In this paper, we propose a patch-wise U-net architecture for the automatic segmentation of brain structures in structural MRI. In the proposed brain segmentation method, the non-overlapping patch-wise U-net is used to overcome the drawbacks of conventional U-net with more retention of local information. In our proposed method, the slices from an MRI scan are divided into non-overlapping patches that are fed into the U-net model along with their corresponding patches of ground truth so as to train the network. The experimental results show that the proposed patch-wise U-net model achieves a Dice similarity coefficient (DSC) score of 0.93 in average and outperforms the conventional U-net and the SegNet-based methods by 3% and 10%, respectively, for on Open Access Series of Imaging Studies (OASIS) and Internet Brain Segmentation Repository (IBSR) dataset.

## 1. Introduction

Segmentation of brain magnetic resonance images (MRI) is a prerequisite to quantifying changes in brain structures [1]. For example, structure atrophy is a well-known biomarker of Alzheimer’s disease and other neurological and degenerative diseases [1]. Among the various modalities such as MRI, computed tomography (CT) and positron emission computed tomography (PET), structural MRI (sMRI) is more preferably used for structural analysis of the brain as it can provide higher contrast images with higher spatial resolution with relatively low health risk associated with cancer, compared to other modalities. Owing to its nature, MRI has been widely used for the segmentation of medical images. Manual segmentation by labeling of pixels or voxels is a significant time-consuming and difficult task. Therefore, it is necessary to develop an automatic segmentation method for brain MRI. For brain MRI segmentation, methods based on pattern recognition algorithms such as the support vector machine [2], random forest [3] and neural network [4], population-specific atlases [5] using demographic factors such as age, gender, ethnicity etc., and the combined methods of both are popularly used. However, the most of currently developed methods require explicit spatial and intensity information. Furthermore, it is required to extract feature vectors from the intensity information for more accurate segmentation performance. In recent years, deep learning-based methods have received significant research attention. In particular, convolutional neural networks (CNNs) [6] have shown high performance in various applications, including handwritten digit recognition, object detection, semantic segmentation etc. Deep learning-based methods usually do not require the extraction of hand-crafted features, thus enabling self-learning of features, while classical machine learning-based methods usually perform feature extraction, such as the Gaussian or Haar-like kernels [7]. One drawback of deep learning-based methods is to require a large amount of training data. In particular, in medical image analysis, it is difficult to obtain such a large amount of labeled training data. Therefore, it is necessary to develop a method that can achieve promising performance, even with a small amount of training data. The patch-based CNNs [7], also called slide-window-based CNNs, are useful in such a scenario because the model can efficiently be learned with a small amount of training data with multi-scale patches, whose sizes are different depending on different modalities such as T1- and T-2 weighted images. However, the training and testing processes of the patch-based CNNs for segmentation take significant computation time because the model needs to run separately for each multi-sized patch. Another approach is to use a data augmentation scheme by applying elastic deformations such as rotation, translation or flipping to the available training data, as in [8–10]. However, the preprocessing for generating a large amount of training data might be computationally complex. The U-net in [8] was initially proposed to segment the neuron structures in an electron microscopic stack and shows good segmentation performance owing to the nature of its U-shape architecture. However, the U-net suffers from a limited memory problem for high resolution of input images because the number of stages of down- and up-sampling increase the feature channels over the resolution of the input images, thus leading to store a number of parameter values at each stage. Moreover, it is known that it is difficult to maintain local details because an entire image is fed into the network. To overcome the problems of the conventional methods, we propose a patch-wise and multi-class U-net architecture for the automatic segmentation of brain MR images. In the proposed method, we first divide an MRI slice into non-overlapping patches to train a U-net model. As the partitioning with individual patches for a slice can better reflect local details by predicting the information for each individual patch, the model can be trained better with the local details in the non-overlapping patches which are made with non-overlapping square partitions in a slice in the proposed method, thus resulting in the higher segmentation performance at efficient computational complexity like the conventional U-net.

The key contribution of the proposed method is the use of individual non-overlapping patches extracted from input slices to train the U-net architecture. The patch-wise splitting of a slice improves the localization accuracy in the MRI tissue segmentation because the trained network is designed for focusing more on local details in a patch. In contrast, randomly selected portions and/or regions from a slices or MRI volume are considered to be patches used for training the model in existing methods [11–16]. This is different from our approach, which divides a whole slice into a number of uniform-sized patches and feed the patches into the model for training. In other words, the network labels each pixel of the uniform-patch. As a consequence, the segmentation task is performed in three stages; a uniform-patch extraction from the input image followed by a pass through the network to obtain the segmentation maps, finally these maps are aggregated to output the final segmented image. High accuracy can be obtained by using the uniform-partitioned patches and eventually entire information of the slices can be used as training data, thus resulting in robust segmentation performances with local detail information. In addition, compared to the previous random patch selection approach which focuses only the selected regions, the proposed method is designed to utilize whole slice information for better segmentation, which can be achieved by using a uniform selection of patches. Moreover, the proposed architecture, compared to the original U-net which can only deal with binary segmentation problems, is able to deal with the multi-class segmentation problem. Moreover, unlike the original U-net, a data augmentation scheme does not need to be applied during the training stage, and the model is therefore trained using only the available training patches in our proposed method. The proposed method shows significant improvement in the Dice similarity coefficient (DSC) [17] and Jaccard index (JI) [18] over the conventional U-net [8] and SegNet-based method [19] for brain structure segmentation. The rest of the paper is organized as follows. In Section 2, related works are described. The proposed method is described in Section 3, and a discussion of experimental results is presented in Section 4. The paper is concluded in Section 5.

## 2. Related studies

Recently, CNNs have been widely used for the segmentation of normal brain structures, e.g., white matter (WM), gray matter (GM), and cerebrospinal fluid (CSF). In [11], two-dimensional patches with a single size from multimodal images, i.e., T1-weighted, T2-weighted and fractional anisotropy images, are used as input for the CNN to segment the three types of structures, namely WM, GM, and CSF, in MR brain images of infants. A method in [11] outperforms the classical machine learning methods, including the support vector machine (SVM) and random forest (RF), in terms of overall DSC scores. From the results, the CNNs for segmentation shows a better performance than the traditional machine learning based methods. This is because the CNNs are capable of providing different weights to each pixel based on the spatial distance to the center pixel, thus resulting in better performance by retaining the spatial information [11]. In [20], fully convolutional networks (FCNs) architecture for segmentation of the brain MRI of infants is proposed and shows improved performance by achieving higher DSC scores, compared to [11]. The model proposed in [20] uses fewer parameters for learning than those of [11], which is beneficial for making the network converge quickly and achieving better performance. In [12], a human brain segmentation method using the patch-wise CNN is proposed with two and three-dimensional patches. The three-dimensional intensity patches take multiple scales of information from the input of the network. Besides, local spatial context is captured by downscaled orthogonal two-dimensional intensity patches and distances to the regional centroid enforce global spatial consistency. In [7], Moeskops et al. recently proposed another patch-wise CNN approach, where different networks are trained with various sized patches and the networks are combined by connecting them to a single soft-max layer. By utilizing the multi-scale patch sizes, the network can also incorporate global spatial consistency with local details. In [19], a pixel-label-based simplified SegNet [21] architecture is proposed. By training the network with a SegNet-based CNN, a DSC score of 0.8 on OASIS dataset is achieved. Table 1 shows the summary of the related works for brain structure segmentation using deep learning. As shown in Table 1, most of the major research work [7, 11, 12] uses the CNN architecture to segment the brain MR images. A number of research work show that the CNN achieves good performance for classification tasks. The CNN can produce the distinct feature maps from a brain MR image, which can work as vectors for classification. A patch-wise 3D U-net for the purpose of performing segmentation of brain tissues was proposed in [13], where the network consists of encoding and decoding layers similar to the conventional U-net, and randomly sampled and overlapped 3D patches (8×24×24) are used for training. Unlike the conventional U-net, a transition layers with convolution operation is used between the encoding and decoding layers to emphasize the impact of feature maps in the decoding layers. Pawel et al. [14] proposed a brain tumor segmentation method using a 3D-CNN, where 3D random patches are obtained and used for training and features extracted by 2D-CNNs (capturing a rich information from a long-range 2D context in three orthogonal directions) are used as an additional input to a 3D-CNN. A brain tumor segmentation method was proposed by using an ensemble of 3D U-Nets with different hyper-parameters trained on non-uniformly extracted patches in [15]. In [16], trained multiple deep neural networks with a 3D U-Net architecture in a tree structure to create segmentations for edema, non-enhancing tumor, and enhancing tumor regions. Furthermore, this 3D segmentation model usually learns from annotations of some slices in the 3D volume and produces a dense 3D segmentation. However, it is widely accepted that it is not well-responsive to user interactions [22, 23], which means the analysis of brain segmentation on 3D space is more difficult than one of the 2D space. In addition, it can be reported in [24–26] that interactive 2D segmentation is more suitable than direct 3D segmentation due to the large inter-slice spacing and motion. Due to the nature of our proposed method, manipulating the individual patches in 3D is significantly computational complex than the case of 2D patches. In addition, compared to random selection of patches which focuses only the selected regions, the proposed method using uniform selection of patches can utilize whole slice information for better segmentation.

**Table 1:** Summary of related studies on brain structure segmentation using deep learning.

Methods	Architecture	Dataset
Moeskops *et al*. [7]	Multi-scale patch-wise CNN	MICCAI 2012
Khagi *et al*. [19]	SegNet	OASIS
Zhang *et al*. [11]	Patch-wise CNN	Private data
Nie *et al*. [20]	FCN	Private data
de Brebission *et al*. [12]	2D/3D patch-wise CNN	MICCAI 2012
Luna *et al*. [13]	3D patch-based CNN	MRBrainS18
Pawel *et al*. [14]	2D/3D Patch-wise CNN	BRATS2017
Xue *et al*. [15]	3D Non-uniform Patch-wise CNN	BRATS2018
Andrew *et al*. [16]	3D Patch-wise CNN	BRATS2017

A semantic-wise CNN architecture, such as SegNet and fully convolutional networks (FCN), is another approach for brain MRI segmentation as described in [19],[20]. It is reported that the SegNet-based segmentation in [19] does not achieve promising segmentation performance, compared to other existing methods. This is because the SegNet tends to lose the neighboring information when up-pooling from low resolution feature maps. In addition, it is more focused on the central slices for training and testing without any results for non-central slices. Despite of advantages, CNN has some drawbacks in segmentation applications because the reconstruction should be done from the vectors in the segmentation process, where we need to not only convert the feature map into a vector but also reconstruct a brain image from this vector. Thus, the U-net has received attention for segmentation owing to its advantages in reconstruction capability over the CNN. It is reported that the U-net can lead to promising results in the segmentation of bio-medical fields [8] due to the capability of preserving the structural integrity of the image which reduces distortion. Nevertheless, the U-net also has some limitations when we want to preserve the local details for segmentation in brain MR images. To overcome the aforementioned drawbacks, our proposed method adopts the non-overlapping patch-wise U-net architecture for brain MRI segmentation. By utilizing non-overlapping patches in a slice, more accurate segmentation performance can be achieved at a similar degree of complexity with the original U-net architecture. In addition, our proposed model can learn from both central and non-central slices, and therefore is able to more accurately segment the brain MR images.

## 3. Proposed method

The SegNet [19] and U-net [8] have popularly been used for segmentation applications. The SegNet [19] architecture largely consists of encoder and decoder parts, whereby the encoding part is used to down-sample the input by using multiple convolution and max-pooling operations, whereas the decoding part is used to up-sample the down-sampled feature maps by using the memorized max-pooling indices from the corresponding encoder feature map and convolution operations. The output of the final decoder is fed to a soft-max classifier to classify each pixel independently. The U-net [8] architecture is very similar to the SegNet architecture, and the main difference between SegNet and U-net lies in the up-sampling part. For the U-net, the feature maps in the decoding path are concatenated with corresponding feature maps in the encoding path during the up-sampling. This property is useful for better localization and for this reason, the U-net was initially proposed particularly for biomedical image segmentation where better localization is crucial for achieving improved performance. Moreover, the up-sampling part also has a large number of feature channels. This allows the network to propagate the context information to higher resolution layers. Table 2 shows the comparative parameter values used for SegNet [19], U-net [8], and the proposed patch-wise U-net architecture, respectively.

**Table 2:** Overall parameter comparison for SegNet, U-net, and proposed path-wise U-net.

Parameters	SegNet [19]	U-net [8]	Proposed patch-wise U-net
Input size	Input image size 208×176	Input image size 256×256	Input image slice 256×256 and divided into four patch size of 128×128
Convolution operation	3×3 convolutions with stride of 1 and padding	3×3 convolutions with stride of 1 and padding	3×3 convolutions with stride of 1 and padding
Activation	ReLU transfer function	ReLU transfer function	ReLU transfer function
Max Operation	2×2 max pooling with stride of 2 and padding	2×2 max pooling with stride of 2 and padding	2×2 max pooling with stride of 2 and padding
Output	Softmax output function	Softmax output function	Softmax output function
Total operations	Total of 8 convolution operations, 2 max-pooling and 2 max-unpooling operations	Total of 15 convolution operations, 3 max-pool and 3 up-conv operations	Total of 15 convolution operations, 3 max-pool and 3 up-conv operations

### 3.1 Architecture of U-net

The U-net architecture was initially proposed for the segmentation of neuronal structures in electron microscopic stacks [8]. The U-net model was an extension of another popular architecture used for segmentation that uses the fully convolutional network (FCN) [27]. The FCN architecture only consists of the encoder path, where the input is down-sampled using successive convolution and max pooling operations, and the final down-sampled feature map is then fed into an activation map to make predictions for individual pixels. On the other hand, the U-net consists of a decoder path, which is almost similar to the encoder path, thus yielding the U-shaped architecture. During decoding or path expansion, the pooling operations are replaced by up-sampling operations. The U-net architecture has shown better performance for segmentation and is much faster than the sliding window-based architecture [28]. However, if the input image size is relatively large, more GPU memory would be required to train the model. In addition, when the architecture takes the entire image as an input, the model is prone to missing the details in certain regions of the image. To overcome the aforementioned limitations, we proposed the non-overlapping patch-wise U-net architecture. The main advantage of our proposed non-overlapping U-net architecture is the patch-wise splitting of a slice obtained from the MRI image, which helps in better localization because the trained network can focus more on local details in a patch.

### 3.2 Proposed segmentation method of the patch-wise U-net

To address the above-mentioned problems, we propose the method of dividing the input image into non-overlapping patches and training the U-net model on these patches. The patches are beneficial for retaining the local information of the image. Moreover, patches with smaller sizes are easier to train than the case of using entire images, as less memory is required for the training and testing. The problem of brain structure segmentation is to segment the brain into multi-class category. Thus, it is difficult to perform the multi-class segmentation using the conventional U-net, where only binary segmentation is performed. To solve the problem, the modification of U-net is proposed to deal with the multi-class segmentation in our proposed method. For this, we have modified the final layer of the U-net, where rather than predicting the binary map for the background and foreground only, the proposed architecture produces the binary map for each of the four classes (background, cerebrospinal fluid, grey matter and white matter).

**Fig 1:**
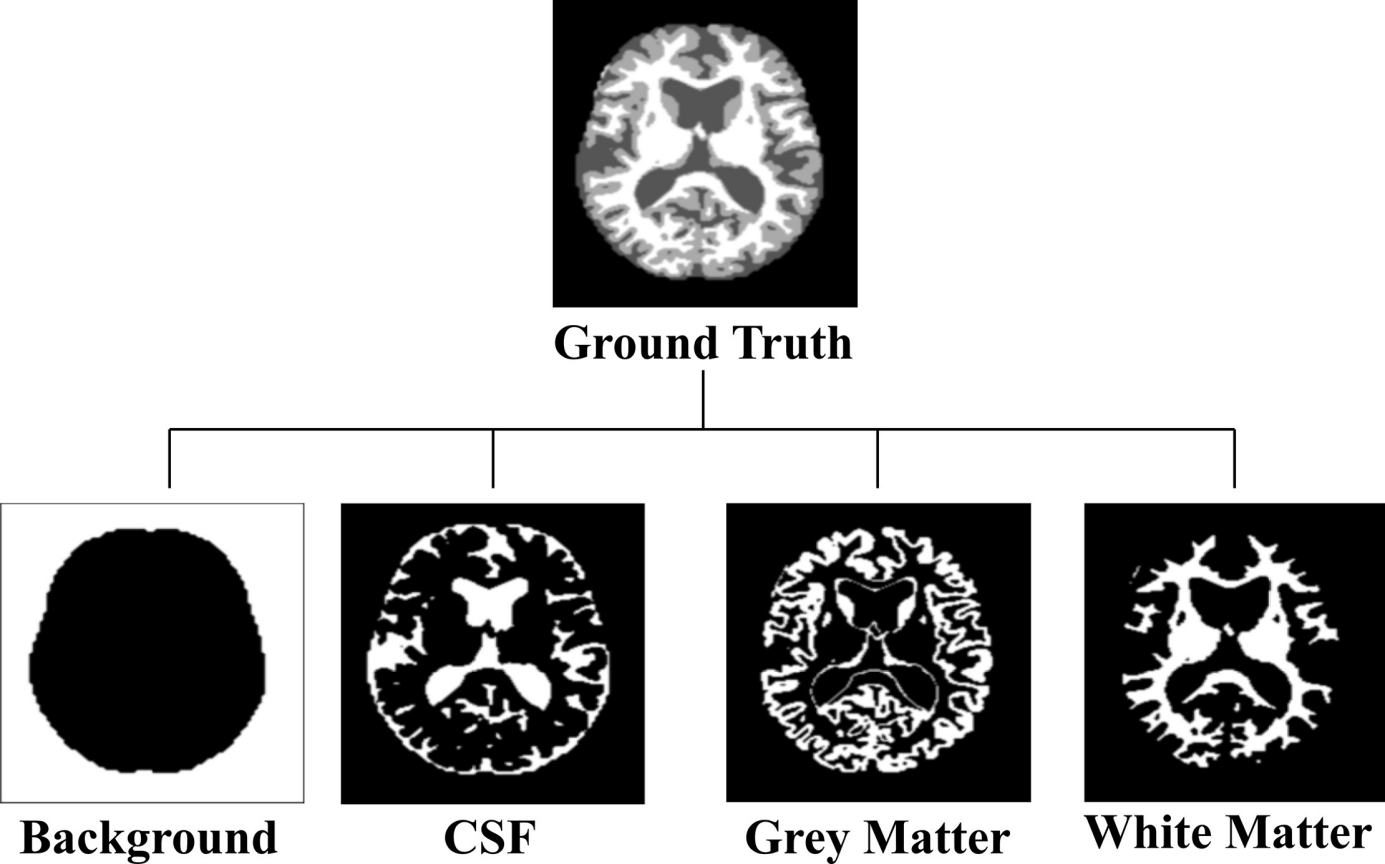
Four different classes of ground truth for segmentation.

The input ground truth segmentation map is also converted into a multi-channel binary segmentation map for each class. Fig 1 shows the multi-channel binary segmentation maps, where the ground truth segmentation map is converted into the background, cerebrospinal fluid (CSF), grey matter (GM), and white matter (WM) binary maps. These four binary maps are treated as a 4-channel target map, which is then fed into the model along with the input for training.

**Fig 2:**
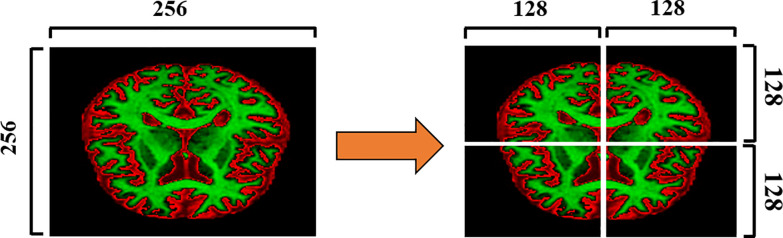
Illustration of a 256×256 pixel-sized input slice divided into four patches each with dimensions of 128×128 pixels.

**Fig 3:**
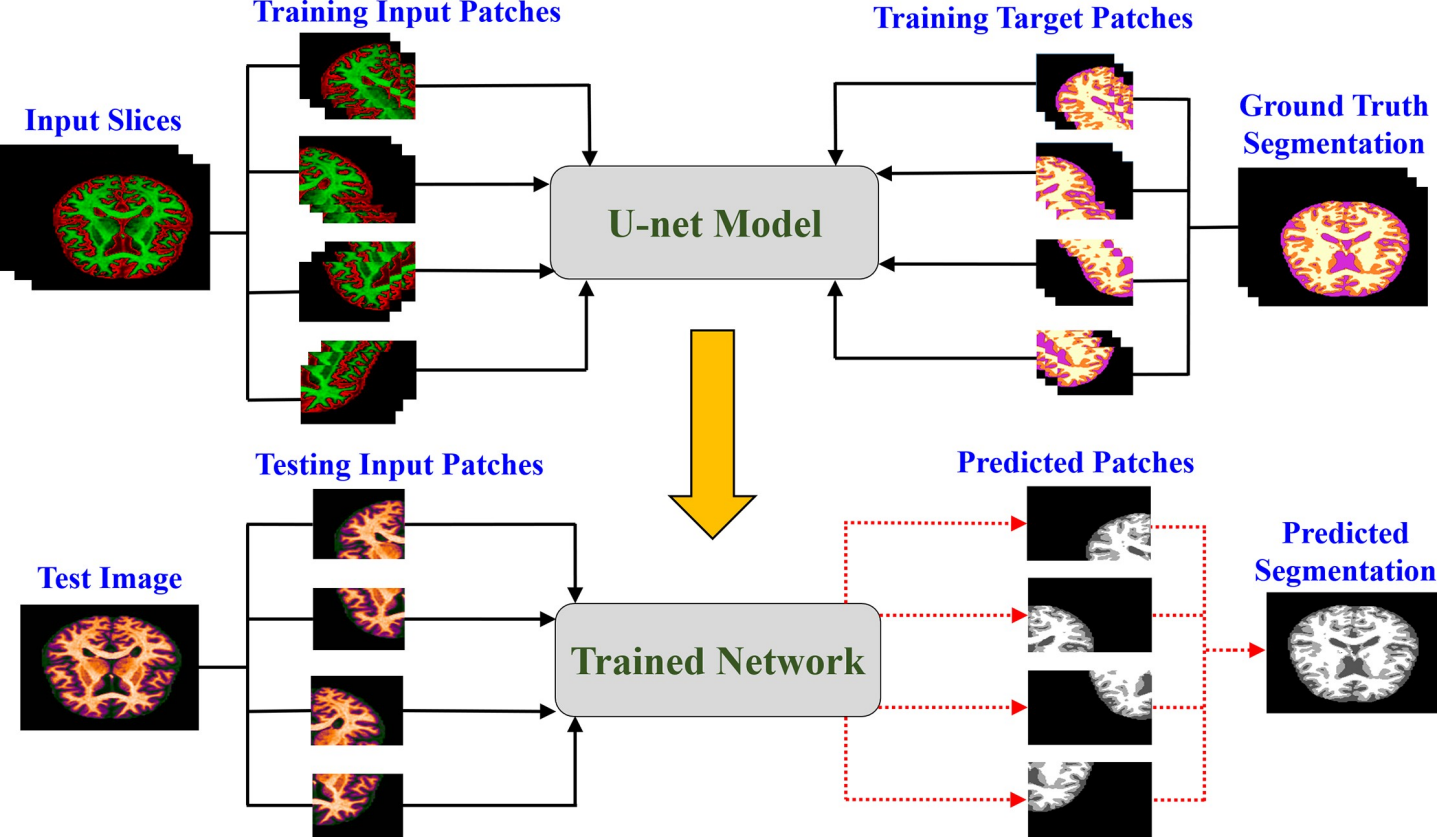
Block diagram of the proposed method.

**Fig 4:**
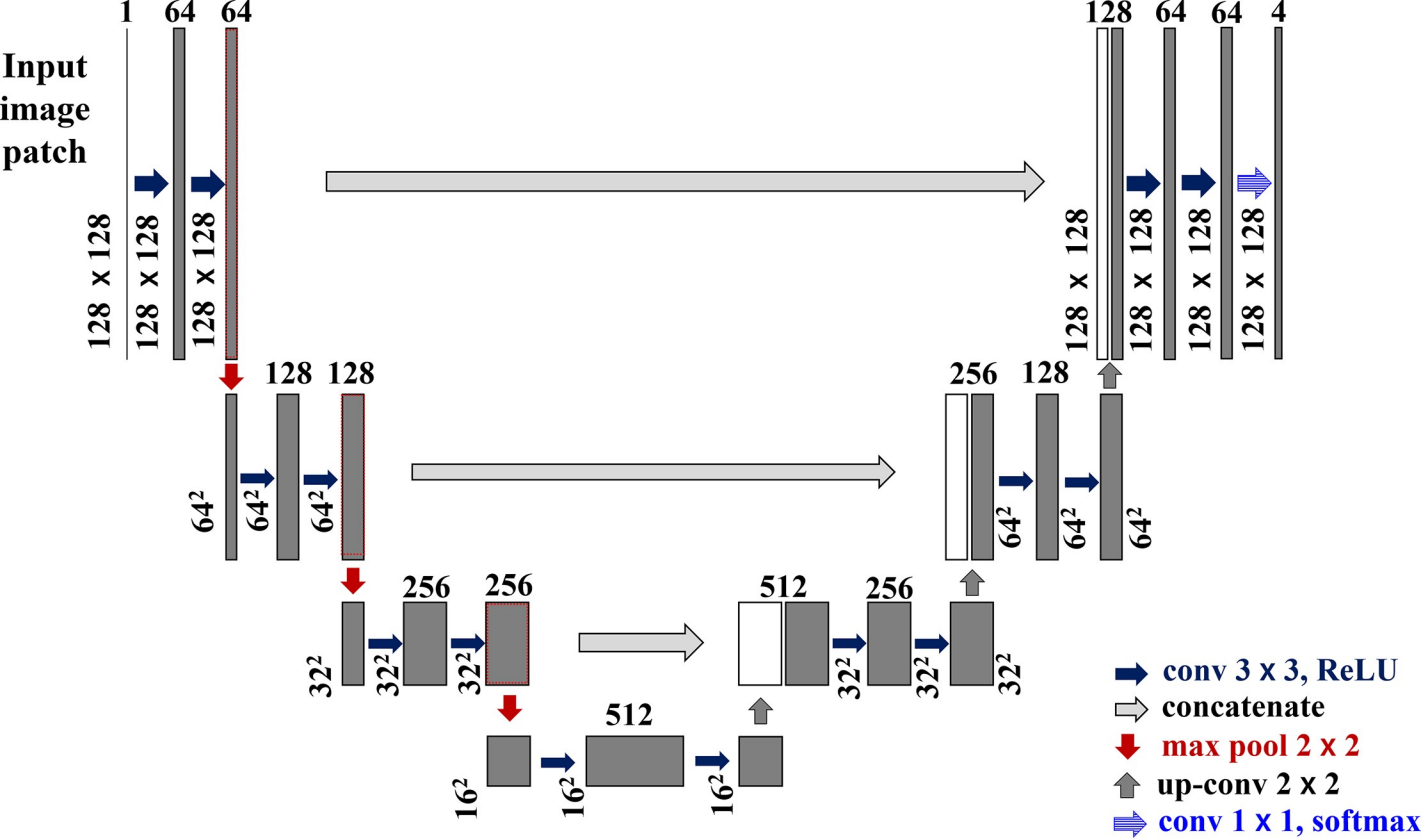
Proposed deep U-net architecture for 128×128 input patch size. Each gray box denotes a multi-channel feature map. The number of channels is denoted on the top of the box. The white boxes represent copied feature maps. The arrows denote the different operations.

Fig 2 shows the splitting of an input slice into a number of patches. The input slices with 256×256 pixel size is divided into four patches with 128×128 pixels. The process is repeated for the ground truth segmentation maps as well. Fig 3 shows a block diagram of the proposed method. During the training stage, slices of each MRI scan and their corresponding ground truth segmentation maps are divided into different patches. As shown in Fig 2, the dimension of the input slice is 256×256, and each slice is split into four different patches. Therefore, the dimension of each resulting patch is half of the input slices in the proposed method. These patches are applied as input to the U-net model for training. The directions of the arrows during the training stage shown in Fig 3 indicate the patches that are fed to the network as input. Fig 4 shows the proposed patch-wise U-net architecture. The 128×128 input patches are input through two consecutive 3×3 convolutions, each followed by a rectified linear unit (ReLU). This is followed by a 2×2 max-pooling operation with stride 2 for down-sampling. After every down-sampling step, the number of feature maps is doubled as suggested in [8]. The process is repeated until the feature map reaches a resolution of 16×16 pixels. This constitutes the contracting path of the network. From here, the expansive path starts with up-sampling of the feature maps followed by a 2×2 convolution (“up-convolution”) that reduces the number of feature channels to half.

**Fig 5:**
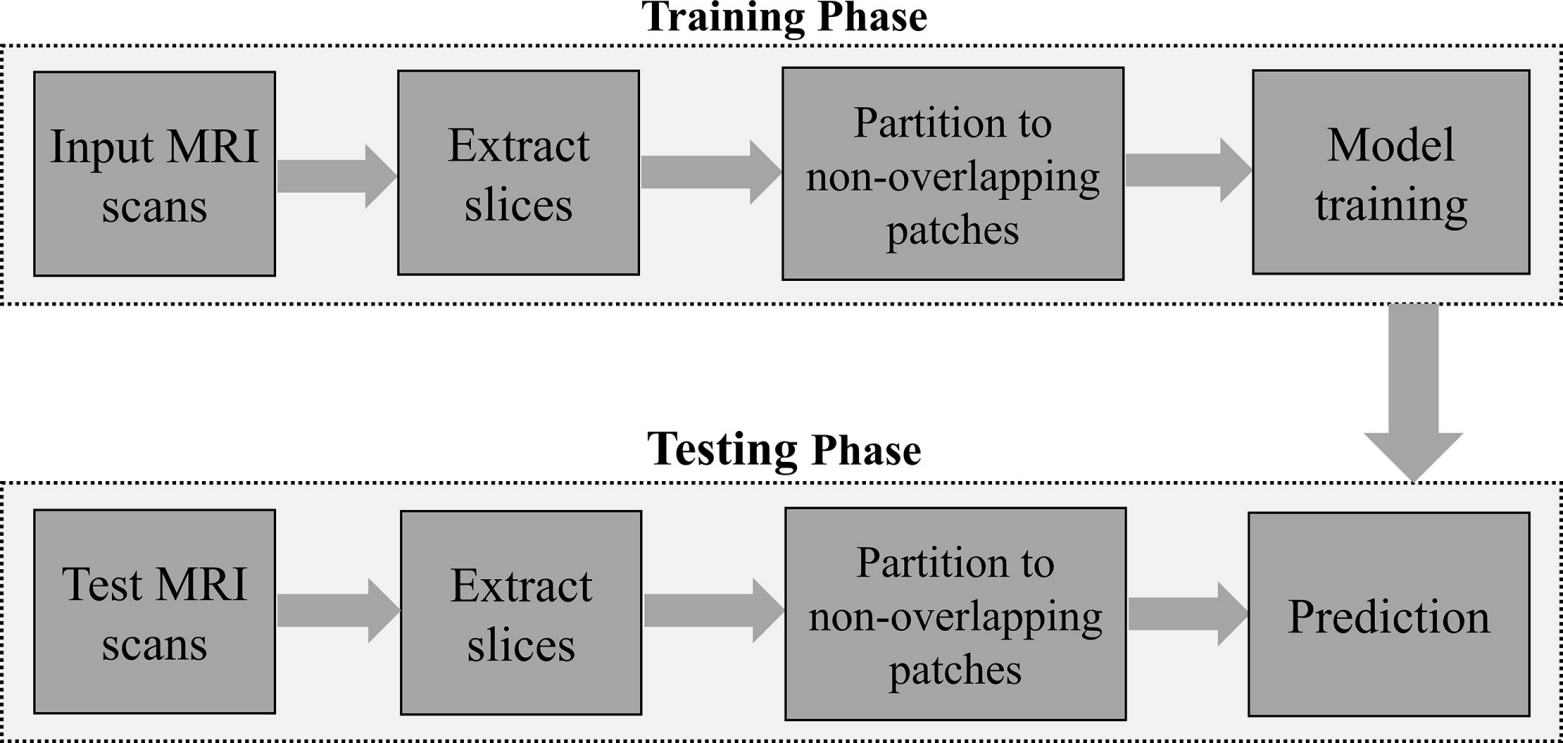
Flowchart of the proposed scheme.

This is followed by a concatenation of the corresponding feature map from the contracting path, and two 3×3 convolutions, each followed by a ReLU. In the final layer, each 64-component feature vector is mapped to the desired number of classes (four in our case), by using the 1×1 convolution. The details of the network architecture are shown in Table 3, and the flow chart of the proposed scheme is shown in Fig 5.

**Table 3:** Parameters of the proposed patch-wise U-net model.

No	Layer name	Type	Output Shape	No. of Parameters	Connected to
1	input_1	Image	128×128×1	0	-
2	conv2d_1	2D Convolution	128×128×64	640	input_1
3	conv2d_2	2D Convolution	128×128×64	36928	conv2d_1
4	max_pooling2d_1	Max Pooling 2D	64×64×64	0	conv2d_2
5	conv2d_3	2D Convolution	64×64×128	73856	max_pooling2d_1
6	conv2d_4	2D Convolution	64×64×128	147584	conv2d_3
7	max_pooling2d_2	Max Pooling 2D	32×32×128	0	conv2d_4
8	conv2d_5	2D Convolution	32×32×256	295168	max_pooling2d_2
9	conv2d_6	2D Convolution	32×32×256	590080	conv2d_5
10	max_pooling2d_3	Max Pooling 2D	16×16×256	0	conv2d_6
11	conv2d_7	2D Convolution	16×16×512	1180160	max_pooling2d_3
12	conv2d_8	2D Convolution	16×16×512	2359808	conv2d_7
13	up_sampling2d_1	Up Sampling 2D	32×32×512	0	conv2d_8
14	conv2d_9	2D Convolution	32×32×256	524544	up_sampling2d_1
15	concatenated_1	Concatenate	32×32×512	0	conv2d_6
					conv2d_9
16	conv2d_10	2D Convolution	32×32×256	1179904	concatenated_1
17	conv2d_11	2D Convolution	32×32×256	590080	conv2d_10
18	up_sampling2d_2	Up Sampling 2D	64×64×256	0	conv2d_11
19	conv2d_12	2D Convolution	64×64×128	131200	up_sampling2d_2
20	concatenated_2	Concatenate	64×64×256	0	conv2d_4
					conv2d_12
21	conv2d_13	2D Convolution	64×64×128	295040	concatenated_2
22	conv2d_14	2D Convolution	64×64×128	147584	conv2d_13
23	up_sampling2d_3	Up Sampling 2D	128×128×128	0	conv2d_14
24	conv2d_15	2D Convolution	128×128×64	32832	up_sampling2d_3
25	concatenated_3	Concatenate	128×128×128	0	conv2d_2
					conv2d_15
26	conv2d_16	2D Convolution	128×128×64	73792	concatenated_3
27	conv2d_17	2D Convolution	128×128×64	36928	conv2d_16
28	conv2d_18	2D Convolution	128×128×4	260	conv2d_17

## 4. Experimental results

For experiments, the model was trained using “Stochastic Gradient Descent (SGD)” with a high momentum rate of 0.99 and a learning rate of 0.001. During the training stage, categorical cross-entropy loss is used to update the learned weights. For initializing the weights, the normalization technique [29] was used. The input slices and their corresponding segmentation maps are divided into four patches and then the resulting patches are used to train the model for the evaluation of the proposed method. The experiments were performed using the Keras [30] framework on Nvidia 1080Ti GPU. The proposed method was evaluated on an Open Access Series of Imaging Studies (OASIS) [31] dataset and International Brain Segmentation Repository (IBSR) [32] datasets. Table 4 shows the details of the OASIS and IBSR dataset. The cross-sectional category for the OASIS dataset consists of T1-weighted MRI scans of 416 subjects. The OASIS dataset is generally used to classify the MRIs into the Alzheimer’s Dementia (AD) or Normal Control (NC) categories, and also includes brain maps for the WM, GM, and CSF. We chose only the first 50 subjects (ID OAS1_0001_MR1 to OAS1_0054_MR1) for the experiment. Out of the selected data, the first 20 MRIs were used for the training and the model was tested on the remaining 30 MRIs. The axial, sagittal and coronal planes of MRI slices are tested for the segmentation of brain MRI. The dimension of the axial scan in the OASIS dataset is 208×176×176 (height×width×slices) and each axial scan consists of 176 slices in total. For the experiment, the original axial scan is resized to a dimension of 256×256×176 by padding 24 pixels of zero to top and bottom of the image and 40 pixels of zeros to left and right of the image. Similarly, the original dimensions of the sagittal (176×208×176) and coronal (176×176×208) scans are resized to dimensions of 256×256×176 and 256×256×208, respectively. We also performed the experiments on the IBSR dataset, which comprises of 18 T1-weighted MRI images of 4 healthy females and 14 healthy males with age ranging from 7 to 71 years. The MRIs in the IBSR are provided after preprocessing such as skull-stripping, normalization and bias field correction. The ground truth is made with manual segmentation by experts with tissue labels as 0, 1, 2 and 3 for background, CSF, GM, and WM, respectively. In our experimentation, the first 12 subjects were used for training, while the model was tested on the remaining 6 subjects. The original axial scans (256×128×256) in the dataset are resized to a dimension of 256×256×256 by zero-padding with 64 pixels to top and bottom of the image to efficiently use the patches in our proposed method. In a similar way, the original dimensions of the sagittal (128×256×256) and coronal (256×256 ×128) are also resized to dimensions of 256×256×256 for the experiments. Thus, every input slice for all planes for the IBSR dataset has the dimension of 256×256 (height×width) during both training and testing. Every input slice across all planes for OASIS and IBSR datasets are adjusted to the dimension of 256×256 (height×width), which allows for using same sized partitioning patches in the proposed method, where the dimensions of each partitioned patches is 128×128 and the patches are input to the proposed model for training and predicted segmentation results can be obtained for the test data. Since any improvement in segmentation results made by data augmentation cannot be observed in our experimental analysis, all of the segmentation results presented in our paper are obtained without using any data augmentation scheme.

**Table 4:** Information for OASIS and IBSR datasets.

	No. of subject	
	OASIS	IBSR
Males	160	14
Females	256	4
Total	**416**	**18**

**Fig 6:**
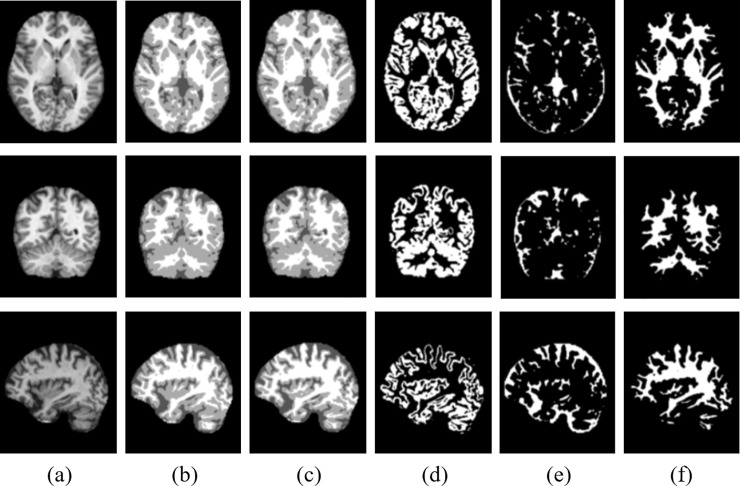
Illustration of segmentation results obtained for our proposed method for axial, coronal and sagittal (top to bottom) using OASIS dataset: (a) Original input images, (b) ground truth segmentation map, (c) predicted segmentation map, (d) predicted GM(binary map), (e) predicted CSF (binary map), and (f) predicted WM (binary map).

**Fig 7:**
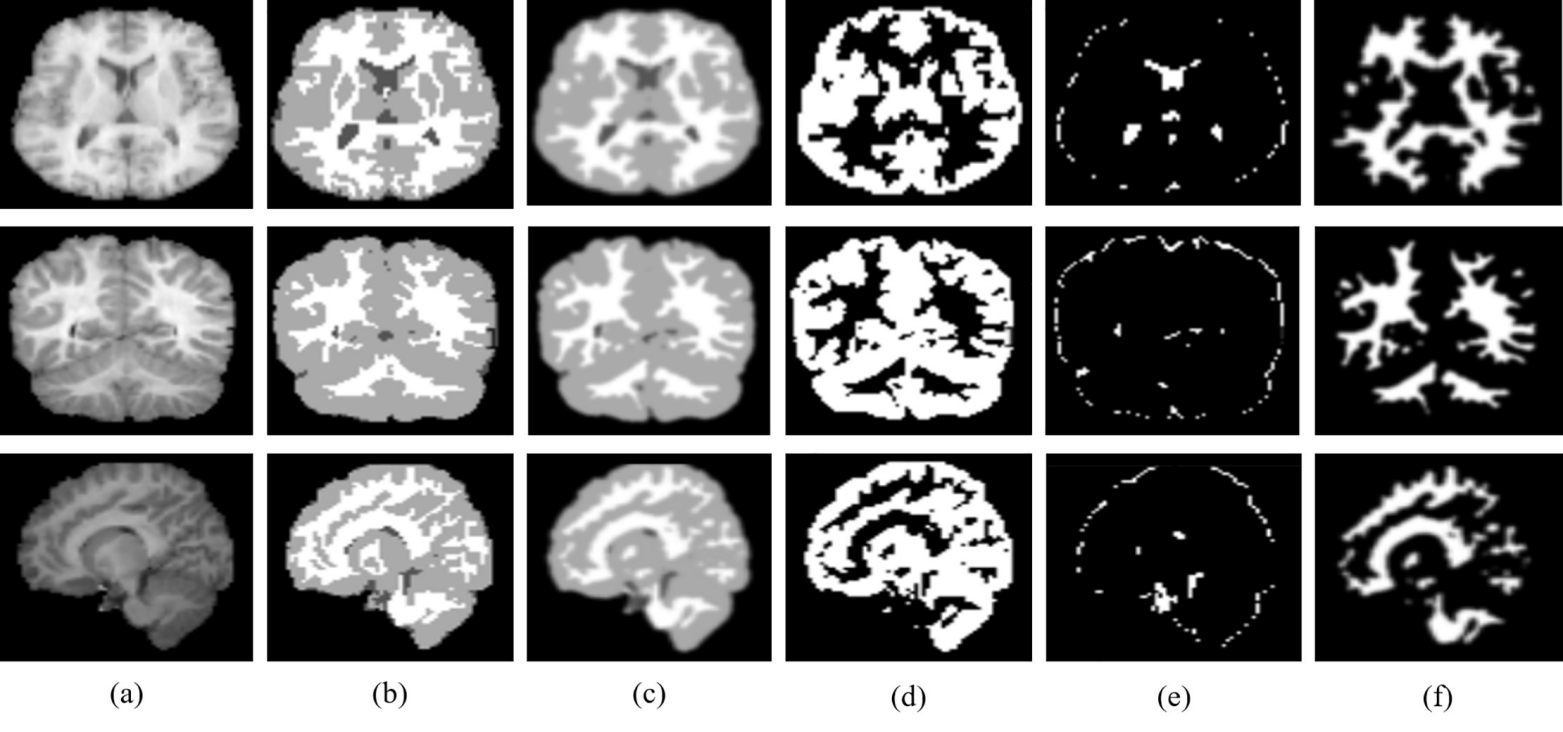
Illustration of segmentation results obtained for our proposed method for axial, coronal and sagittal (top to bottom) using IBSR dataset: (a) Original input image, (b) ground truth segmentation map, (c) predicted segmentation map, (d) predicted GM (binary map), (e) predicted CSF (binary map), and (f) predicted WM (binary map).

Fig 6 and Fig 7 show the segmentation results for axial, coronal and sagittal planes of the OASIS and IBSR datasets, respectively. The first and second columns in Fig 6 and Fig 7 show original images and the ground truth segmentation maps corresponding to each of the given slices, respectively. The third column in Fig 6 and Fig 7 shows the predicted segmentation map using the proposed method. In the fourth, fifth and sixth columns, binary prediction maps for GM, CSF and WM are shown respectively. It can be observed in Fig 6 and Fig 7 that the proposed method achieves well-segmented results on both datasets.

We also performed experiments by implementing the U-net [8], SegNet [19], and proposed non-overlapping patch-wise U-net for comparison. Table 5 shows the performance comparisons between the proposed method, conventional U-net [8] and SegNet [19]. To objectively evaluate the performances of the methods, the Dice similarity coefficient (DSC) [17] and Jaccard index (JI) [18] were used, which are commonly used to evaluate the performance of segmentation algorithms. The metrics are used to compute the similarity of two sample sets for segmentation and indicate how closely the predicted segmentation map matches the ground truth segmentation map. The JI and DSC scores between the ground truth segmentation map I and the predicted segmentation map I’ are defined as (1) and (2) [17–18].

$$JI(I,I^{\prime })=\frac{\left|I\cap I^{\prime }\right|}{\left|I\cup I^{\prime }\right|}$$ (1)

$$DSC(I,I^{\prime })=\frac{2|I\cap I^{\prime }|}{\left|I|+|I^{\prime }\right|}$$ (2)

where |.| represents the cardinality of the set. Furthermore, the Hausdorff distance (HD) [33] between ground truth segmentation map *I* and the predicted one *I*’ is measured for GM, WM and CSF. The HD is the maximum distance of set to the nearest point in the other set and defined as (3).

$$d(I,I^{\prime })=max\left\{\underset{a\in I}{max}\;\underset{b\in I^{\prime }}{min}|b-a|,\underset{b\in I^{\prime }}{max}\;\underset{a\in I}{min}|a-b|\right\}$$ (3)

where *a* and *b* are points of sets *I* and *I*′, respectively. In other words, the HD between *I* and *I*′ is the smallest value *d* such that every point of *I* has a point of *I*′ within distance *d* and every point of *I*′ has a point of *I* within distance *d* [33].

**Table 5:** Segmentation result comparisons between the proposed, the conventional U-net, and SegNet based methods for OASIS and IBSR datasets (DSC: Dice Similarity Coefficient, JI: Jaccard Index, MSE: Mean Square error, GM: Grey Matter, WM: White Mater, CSF: Cerebrospinal Fluid, HD: Hausdorff distance).

	OASIS Dataset									
	Axial plane									
Methods	WM			GM			CSF			MSE
	DSC (%)	JI (%)	HD	DSC (%)	JI (%)	HD	DSC (%)	JI (%)	HD	
SegNet[19]	0.87±0.017	0.77±0.021	5.09±0.18	0.84±0.014	0.72±0.011	5.7±0.53	0.80±0.045	0.67±0.063	4.9±0.47	0.023
U-net[8]	0.93±0.012	0.87±0.018	4.40±0.15	0.90±0.023	0.82±0.034	4.3±0.24	0.88±0.045	0.80±0.056	4.6±0.26	0.017
**Proposed method**	**0.94**±**0.008**	**0.89**±**0.014**	**3.28**±**0.31**	**0.93**±**0.011**	**0.87**±**0.021**	**3.9**±**0.11**	**0.93**±**0.013**	**0.88**±**0.035**	**3.6**±**0.05**	**0.008**
	**Coronal plane**									
SegNet[19]	0.82±0.054	0.69±0.046	5.4±0.35	0.78±0.038	0.64±0.049	4.6±0.58	0.74±0.067	0.61±0.091	4.6±0.43	0.035
U-net[8]	0.93±0.015	0.87±0.020	4.14±0.21	0.92±0.018	0.85±0.028	4.2±0.34	0.89±0.032	0.82±0.036	4.1±0.37	0.015
**Proposed method**	**0.95**±**0.006**	**0.91**±**0.012**	**3.16**±**0.22**	**0.94**±**0.013**	**0.88**±**0.023**	**3.3**±**0.18**	**0.92**±**0.020**	**0.85**±**0.035**	**3.2**±**0.19**	**0.009**
	**Sagittal Plane**									
SegNet[19]	0.82±0.029	0.69±0.034	7.2±0.43	0.80±0.046	0.67±0.057	5.9±0.29	0.77±0.069	0.63±0.084	6.3±0.52	0.028
U-net[8]	0.92±0.019	0.86±0.024	4.34±0.38	0.91±0.020	0.83±0.027	5.2±0.23	0.88±0.024	0.81±0.029	4.4±0.34	0.019
**Proposed method**	**0.94**±**0.014**	**0.90**±**0.025**	**4.20**±**0.15**	**0.93**±**0.009**	**0.87**±**0.016**	**4.6**±**0.10**	**0.93**±**0.017**	**0.88**±**0.043**	**3.3**±**0.11**	**0.009**
	**IBSR Dataset**									
	**Axial plane**									
SegNet[19]	0.72±0.036	0.65±0.042	6.51±0.65	0.75±0.049	0.67±0.058	6.53±0.91	0.68±0.099	0.59±0.095	6.96±0.46	0.039
U-net[8]	0.89±0.022	0.81±0.034	5.14±0.51	0.91±0.017	0.85±0.023	4.87±0.51	0.84±0.065	0.75±0.079	5.24±0.31	0.023
**Proposed method**	**0.91**±**0.031**	**0.82**±**0.047**	**4.54**±**0.23**	**0.93**±**0.029**	**0.86**±**0.038**	**4.33**±**0.13**	**0.85**±**0.057**	**0.77**±**0.081**	**4.14**±**0.18**	**0.016**
	**Coronal plane**									
SegNet[19]	0.70±0.061	0.62±0.051	6.32±0.82	0.73±0.037	0.65±0.062	6.21±0.84	0.66±0.054	0.57±0.086	6.84±0.75	0.032
U-net[8]	0.88±0.035	0.79±0.034	5.45±0.67	0.90±0.014	0.83±0.056	5.17±0.38	0.83±0.012	0.76±0.043	5.54±0.47	0.021
**Proposed method**	**0.90**±**0.018**	**0.81**±**0.079**	**4.61**±**0.21**	**0.92**±**0.024**	**0.86**±**0.047**	**4.56**±**0.19**	**0.83**±**0.078**	**0.78**±**0.066**	**4.73**±**0.25**	**0.019**
	**Sagittal Plane**									
SegNet[19]	0.71±0.043	0.63±0.039	6.49±0.61	0.74±0.073	0.66±0.059	6.36±0.76	0.65±0.083	0.54±0.092	6.99±0.41	0.041
U-net[8]	0.86±0.029	0.78±0.062	5.75±0.37	0.89±0.036	0.81±0.041	5.77±0.21	0.80±0.071	0.73±0.019	5.83±0.15	0.026
**Proposed method**	**0.89**±**0.012**	**0.80**±**0.032**	**4.89**±**0.014**	**0.91**±**0.002**	**0.84**±**0.023**	**5.42**±**0.06**	**0.81**±**0.040**	**0.75**±**0.041**	**4.98**±**0.09**	**0.021**

We also further analyzed segmentation performance in terms of the mean square error (MSE) [34], which is an average square difference between the original and predicted values and computed as (4).

$$MSE=\frac{1}{MN}\sum_{i=0}^{M-1}\sum_{j=0}^{N-1}{\left(I_{i,j}-I_{i,j}^{\prime }\right)}^{2}$$ (4)

where *M* and *N* are the width and height of the image, *I*_*i*,*j*_ and $I_{i,j}^{\prime }$ are the original and predicted segmentation maps, and *i* and *j* are the pixel indices, respectively.

**Fig 8:**
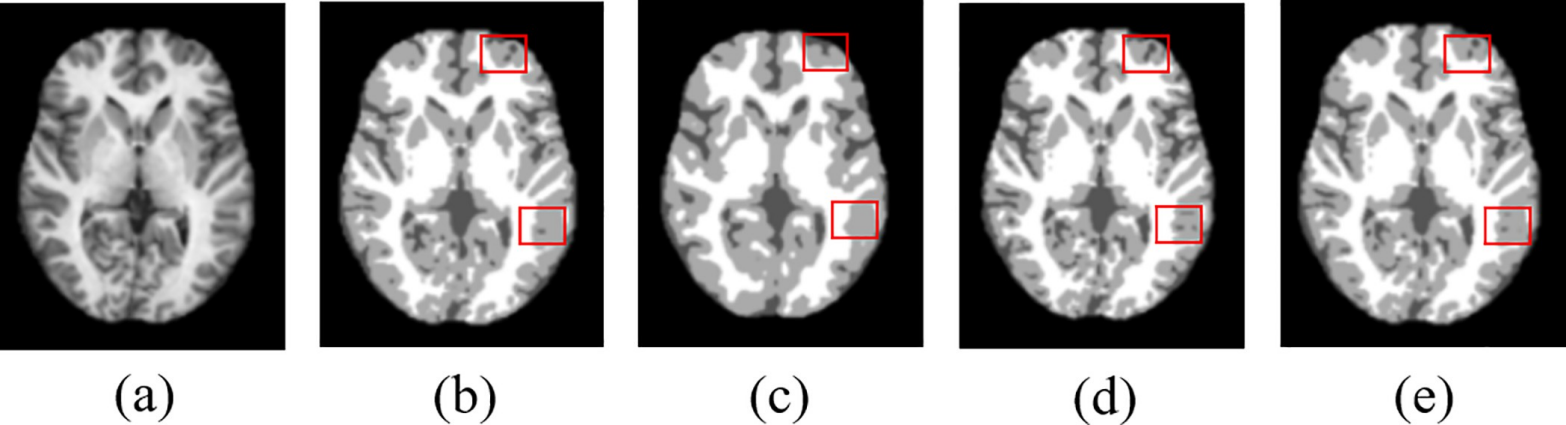
Segmentation results for existing methods and the proposed method: (a) original input image, (b) ground-truth segmentation map, and segmentation maps generated by (c) SegNet, (d) U-net, and (e) Proposed method.

As shown in Fig 8, the proposed method produces the best segmentation results. Compared to the results of the other segmentation methods, the quality of the segmentation map generated by the proposed method (Fig 8(E)) is clearly superior. One can observe that the segmentation results of the U-net and SegNet architectures lack fine details compared to those of the proposed method, as indicated by the red squares in Fig 8(C) and Fig 8(D). The performances of the segmentation are shown in terms of the DSC, JI and HD score in Table 5 for OASIS and IBSR datasets. As shown in Table 5, the proposed non-overlapping patch-wise segmentation method outperforms the conventional U-net [8] and SegNet-based method [19] in terms of DSC, JI and HD. It is noted that the conventional U-net [8] is the case of using a whole image as the input whereas the proposed method utilizes the non-overlapping patches based on the U-net architecture. As discussed in section 3, experimental results show that the conventional U-net does not lead to promising segmentation performance due to lack of local details whereas our proposed method shows significantly higher performances than those of the U-net owing to the effect of the non-overlapping patches. Our proposed method also outperforms the SegNet-based method. In particular, the SegNet-based method shows significantly lower performance than the other two methods. This is due to the fact that the SegNet tends to lose the neighboring information when up-pooling from low resolution feature maps. This is attributed to the fact that the conventional U-net and the proposed method use a more optimal up-sampling “up-convolution” (also known as “transpose convolution”) method. As the slices are divided into patches and the predictions for each patch are made separately in the proposed method, local information can be preserved in a better way, thus resulting in better segmentation performance, compared to the conventional U-net model which uses whole slices as input.

The proposed method achieves the best results in terms of DSC, JI, HD and MSE for all planes and shows the consistent results for OASIS and IBSR datasets. It is noted that unlike the results on the OASIS dataset, mean DSC values of CSF do not show significant improvement over the existing methods on the IBSR dataset because original ground-truth annotations in the IBSR do not contain sulcal parts of CSF tissue unlike GM [35]. There are several studies [36,37] using the IBSR datasets with labeled sulcal CSF (SCSF) voxels to reduce the differences between segmentation masks and ground-truth labels. However, for fair and stable comparison, we performed the experiments using the original IBSR dataset without additional annotation and compared our method with other methods under the same experimental conditions. In addition, the maximum standard deviations for DSC, JI and HD are 0.078, 0.087 and 0.31, respectively, which are close to the mean values and indicates that the pixel predicted values are fitted well to the ground truth values without much data variation.

**Fig 9:**
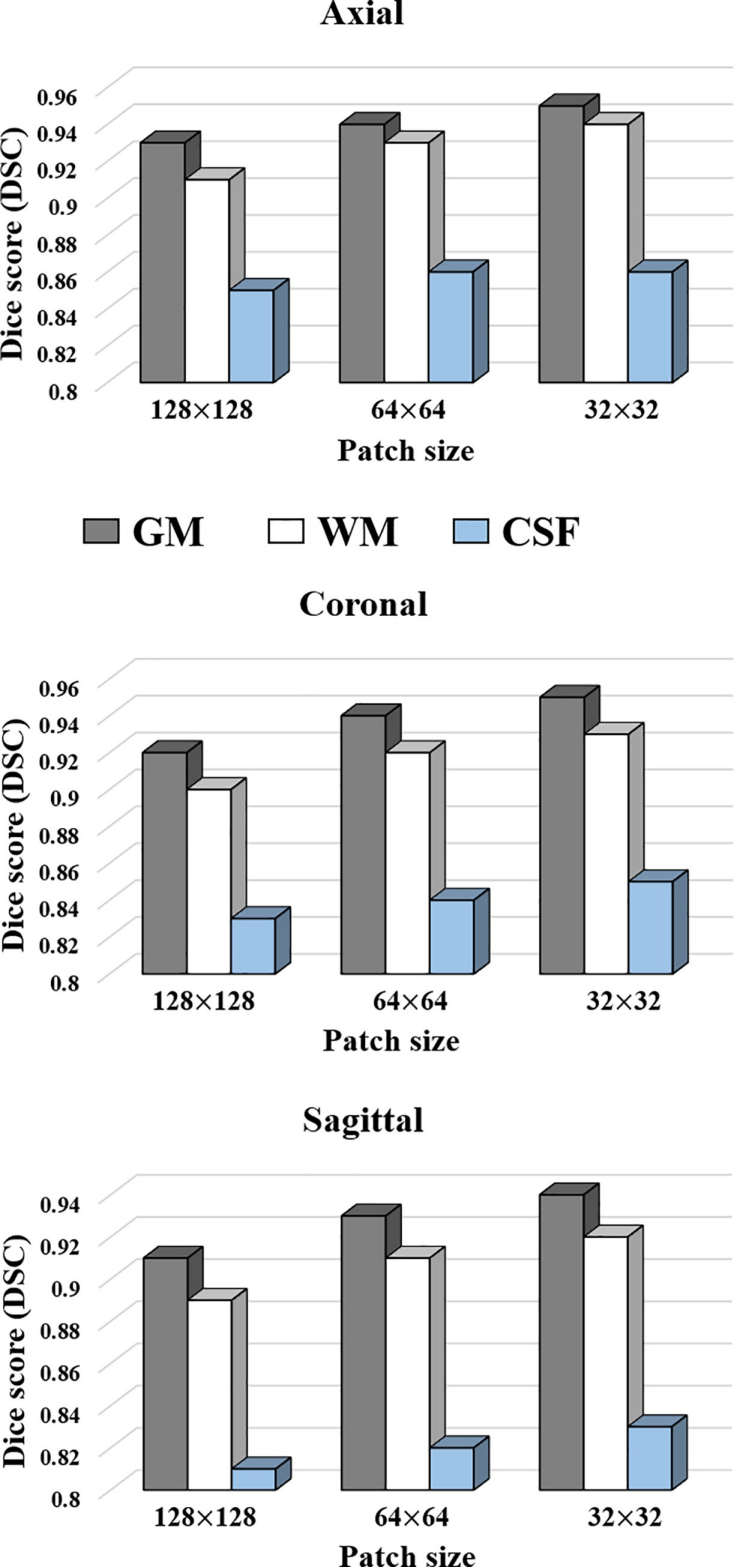
DSC scores with respect to different patch sizes for OASIS dataset.

We also investigated the effect of the patch sizes in terms of running time and segmentation performance. The experiments were performed on OASIS dataset for three different patch sizes (128 × 128, 64 × 64 and 32 × 32). The graph for segmentation performance in DSC with respect to different patch sizes are as shown in Fig 9. It can be observed that the smaller patch sizes result in better performance in terms of the DSC score. This is because a smaller patch size can produce more training data for the network to train. Moreover, the local regions will be reconstructed more precisely.

Table 6 shows the execution time according to the patch size in the proposed method. When the patch size is 128×128, it takes only 30*s* per subject, whereas it takes 65*s* for 32 ×32 patches. Therefore, it is concluded that the 128×128 patch size presents a decent tradeoff between the DSC score and computational time taken to predict a single subject, based on the results of Table 6 and Fig 9.

**Table 6:** Runtime time performance with respect to a different patch size for U-net model to predict the complete test subject.

Patch size (pixels)	Time (*s*)
128 × 128	30
64 × 64	47
32 × 32	65

To evaluate the effectiveness of the non-overlapping patches, we compared the performance of the proposed method with the conventional U-net and overlapping patch-wise U-net. As discussed earlier, the whole slices are used for training and testing as input in the conventional U-net while partitioned patches are used for the overlapping and the proposed non-overlapping cases. Table 7 shows the experimental setups and results for three cases under consideration. For comparison with the conventional U-net [8], the proposed method achieves higher DSC and JI values by 0.3 and 0.7, respectively. For the comparison with the overlapping U-net, overlapping patches with the same size used in the proposed method (but a slide of 8 pixels) are taken. The underlying reason for using a stride of 8 pixels is that a pixel stride of less than 8 pixels shows almost identical segmentation results as overlapping patches with a small stride difference might share similar information. Furthermore, as the pixel stride is smaller, the number of patches increases, which causes increased computational complexity. For instance, starting with a stride of 1 pixel, each slice will create 258 patches. Each subject with 176 slices (axial plane) will have 45,408 (176×258) patches and multiplied by 20 subjects (for training), yielding a total number of 908,160 patches; training process using such a huge number of patches at a time leads to increased computational time. In contrast, if we take 8 pixels stride, it will create overall 112,640 (20×176×32) patches during the training stage. Note that this significantly reduced number of patches shows identical results as the case of choosing a stride of 1 pixel. For this reason, an optimal stride size of 8 pixels is chosen for our experimental study.

**Table 7:** Overall parameter comparison table used for automatic segmentation brain MR images based on the U-net model.

No.	Parameters	U-Net[8]	Overlapping patch-wise U-Net	Proposed method
1	Input size	256×256	128×128	128×128
2	Training set	20 subjects	20 subjects	20 subjects
3	Testing set	30 subjects	30 subjects	30 subjects
4	Slice extraction (for axial)	176 slices from each subject	176 slices from each subject	176 slices from each subject
5	# of patches (for 1 slice)	1	32 (Stride of 8 pixels)	4
6	Computational time	3 *hours*	35 *hours*	4 *hours*
7	DSC (OASIS dataset)	0.90	0.94	0.93
8	JI (OASIS dataset)	0.81	0.89	0.87

Although the classification accuracies show almost identical results of 0.94 for DSC and 0.89 for JI, respectively, the output of the predicted image cannot be reconstructed accurately. This is because of multiple convolution operation over the same pixel elements. Moreover, the overlapping patch-based U-net requires significantly more computation time because the network should be trained separately for each overlapping patch. The overall computation complexity of overlapping patch-wise U-net requires 35 hours to train and test the images under our experimental setup. On the other hand, the proposed method only takes 4 hours.

**Fig 10:**
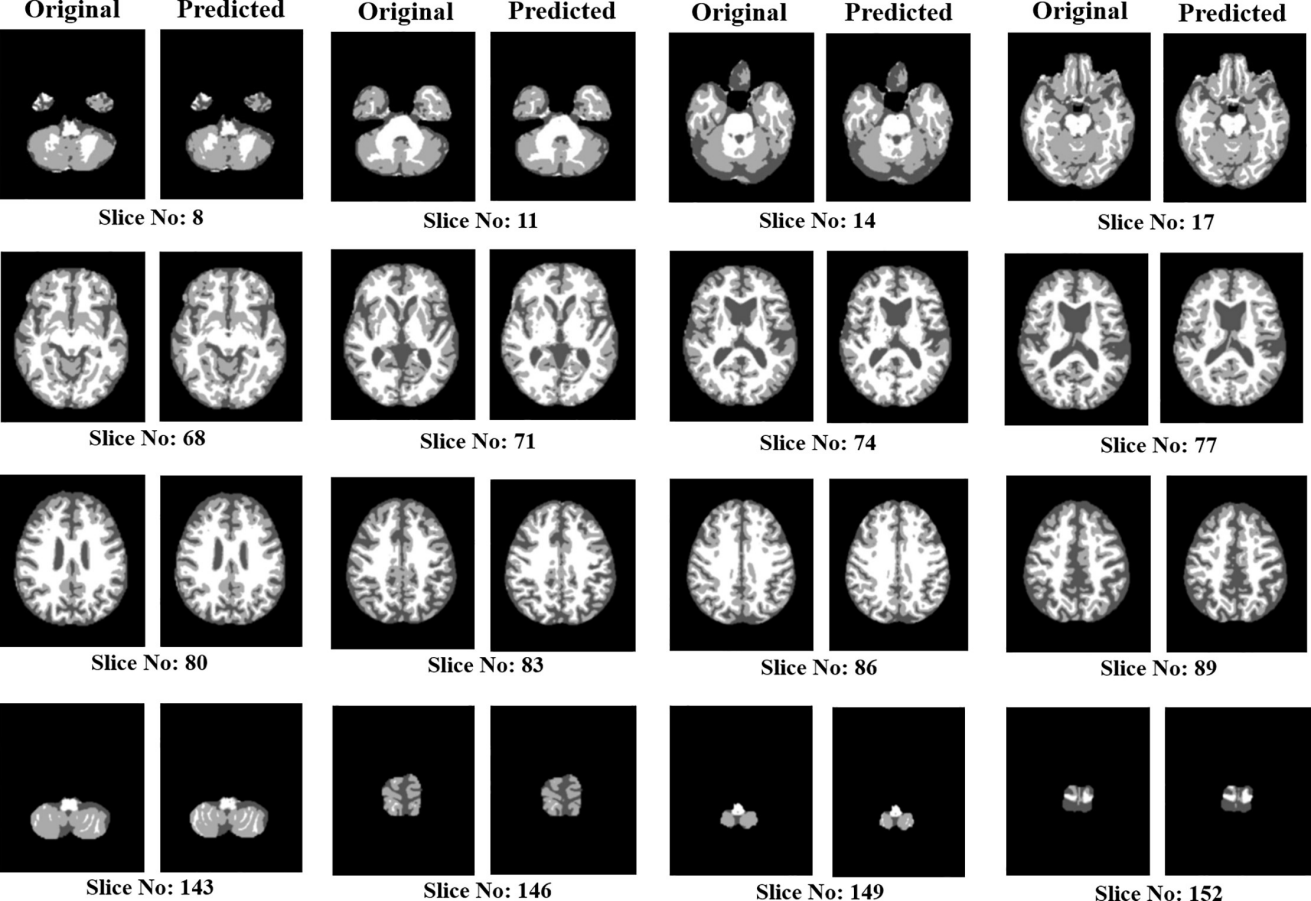
Results of predicted images along with original images based on central and non-central slices.

In order to investigate the effect of non-central slices for the result, the segmentation experiments were performed using non-central slices. Fig 10 shows the results of both central and non-central slices. Here, we can observe that the non-central slices such as 8, 11, 14, 17,143, 146, 149 and 152 contain less information compared to the other central slices. Although non-central slices contain less information, the proposed method is capable of accurately segmenting the predicted images with respect to the original images. The segmentation results of the proposed method are shown in Fig 10.

For training, some slices at the side lobes do not contain much useful information [38] for segmentation and a slice shares almost the same information with neighboring slices. Hence, by excluding these non-informative slices and reducing the repetitive training of the consecutive slices, we extracted 48 slices with an interval of 3 slices, which contains both central slices (i.e., slices with more information) and non-central slices (i.e., slice with less information) for training. Table 8 shows the experimental results for the comparison between the cases of using 48 slices and all slices. As shown in Table 8, performance differences between the two cases are almost negligible even if using only 48 slices takes half complexity compared to the case of using all slices. From the results, it would be much beneficial to use selected slices for training without the degradation of segmentation performance while reducing computational complexity.

**Table 8:** Comparison of segmentation results between the cases of using 48 slices and all slices in the training.

Plane	Parameter	CSF		GM		WM		Execution time	
		Selected 48 slices	All slices	Selected 48 slices	All slices	Selected 48 slices	All slices	Selected 48 slices	All slices
Axial	DSC	0.92	0.93	0.93	0.93	0.94	0.94	59 minutes	4 hours
	JI	0.85	0.88	0.87	0.87	0.89	0.89		
Coronal	DSC	0.91	0.92	0.93	0.94	0.94	0.95	1.5 hours	4.5 hours
	JI	0.84	0.85	0.88	0.88	0.89	0.91		
Sagittal	DSC	0.92	0.93	0.92	0.93	0.93	0.94	59 minutes	4 hours
	JI	0.85	0.88	0.86	0.87	0.88	0.90		

To investigate the impact on the random selection of the subset dataset, the additional experiments were performed using randomly selected datasets by Table 9.

**Table 9:** Training and test dataset for investigating the impact of a random selection of the subset dataset.

No. of test set	Training (Subject #)	Test (Subject #)
Testset0	0–20	21–50
Testset1	100–120	200–230
Testset2	300–320	21–50

Table 10 shows the segmentation results for randomly selected sub-datasets of the OASIS datasets. As shown in the table, the proposed method shows almost identical performances with Table 5 of the revised manuscript. It indicates that our proposed method shows robust segmentation performances regardless of the construction approach of datasets.

**Table 10:** Segmentation results for randomly selected datasets of the OASIS datasets.

Sets	Parameter	GM	WM	CSF
Testset0	DSC	0.93	0.94	0.93
	JI	0.87	0.89	0.88
Testset1	DSC	0.91	0.92	0.94
	JI	0.84	0.85	0.88
Testset2	DSC	0.94	0.95	0.93
	JI	0.89	0.91	0.88

## 5. Conclusions

In this paper, we have shown that, by dividing the input slices and the corresponding segmentation maps of brain MRI and training the U-net model on these, one can achieve segmentation performance that is better than that of previously proposed methods. The proposed patch-wise U-net architecture makes predictions for the input patches individually, and hence, the local spatial information is better retained. Moreover, the proposed model also has the ability to make predictions for multi-class segmentation, as opposed to the conventional U-net model, which was proposed to deal with binary segmentation problem. Even though our method has a limitation of increasing the computational complexity in training, it is negligible, compared to the conventional U-Net model, considering significantly improved segmentation performances over other state-of-the-art methods. Our method also shows significant improvement in terms of metrics such as the Dice similarity coefficient and Jaccard index, for segmentation of the brain MRI to CSF, GM, and WM regions with an overall DSC score of 0.93, which shows an improvement of approximately 3% over the conventional U-net and significant improvement over the SegNet-based approach by more than 10%.
