# Correction: Automatic segmentation of brain MRI using a novel patch-wise U-net deep architecture

**DOI:** 10.1371/journal.pone.0246105

**Published:** 2021-01-22

**Authors:** The PLOS ONE Staff

There are errors in the Funding statement. The publisher apologizes for the errors. The correct Funding statement is as follows: This work was supported by the National Research Foundation of Korea (NRF) grant funded by the Korea government under Grant NRF-2019R1A4A1029769 and Grant NRF-2019R1I1A3A01058959.
