# Correction: Automatic segmentation of brain MRI using a novel patch-wise U-net deep architecture

**DOI:** 10.1371/journal.pone.0264231

**Published:** 2022-02-14

**Authors:** Bumshik Lee, Nagaraj Yamanakkanavar, Muhammad Ammar Malik, Jae Young Choi

Muhammad Ammar Malik is not included in the author byline. Muhammad Ammar Malik should be listed as the third author, and their affiliations are the Department of Information and Communications Engineering, Chosun University, Gwangju, Republic of Korea and the Department of Informatics, University of Bergen, Bergen, Norway.

The correct citation is: Lee B, Yamanakkanavar N, Malik MA, Choi JY (2020) Automatic segmentation of brain MRI using a novel patch-wise U-net deep architecture. PLoS ONE 15(8): e0236493. https://doi.org/10.1371/journal.pone.0236493

The contributions of this author are as follows: Formal analysis, Investigation, Methodology, Software, Writing–original draft

The complete author contributions are therefore as follows:

Conceptualization: BL, JYC

Formal analysis: BL, MAM, JYC

Funding acquisition: BL

Investigation: NY, MAM, JYCMethodology: BL, NY, MAM

Project administration: BL

Software: BL, NY, MAM

Supervision: JYCValidation: NY, JYC,

Visualization: NY, JYC

Writing–original draft: BL, MAM, JYC

Writing–review & editing: BL, NY, JYC
